# Management of chronic renal allograft dysfunction and when to re-transplant

**DOI:** 10.1007/s00467-018-4000-9

**Published:** 2018-07-23

**Authors:** Richard J. Baker, Stephen D. Marks

**Affiliations:** 1 Renal Unit, Lincoln Wing, St. James’s University Hospital, Beckett Street, Leeds, LS9 7TF UK; 2 Department of Paediatric Nephrology, Great Ormond Street Hospital for Children NHS Foundation Trust, London, UK; 3 University College London Great Ormond Street Institute of Child Health, London, UK

**Keywords:** Renal transplantation, Renal allograft failure, Re-transplantation

## Abstract

Despite the advances in renal transplantation over the last decades, chronic allograft dysfunction remains the largest concern for patients, their families, clinicians and other members of the multi-disciplinary team. Although we have made progress in improving patient and renal allograft survival within the first year after transplantation, the rate of transplant failure with requirement for commencement of dialysis or re-transplantation has essentially remained unchanged. It is important that paediatric and adult nephrologists and transplant surgeons, not only manage their patients and their renal transplants but provide the best chronic kidney disease management during the time of decline of renal allograft function. The gold standard for patients with Stage V chronic kidney disease is to have pre-emptive living donor transplants, where possible and the same is true for healthy renal transplant recipients with declining renal allograft function. The consideration for children and young people as they embark on their end-stage kidney disease journey is the risk-benefit profile of giving the best immunologically matched and good quality renal allografts as they may require multiple renal transplantation operations during their lifetime.

## Introduction

Transplant professionals often exhort their colleagues to consider pre-emptive renal transplantation as the primary form of renal replacement therapy, preferably with a living donor. It would seem intuitive that the same individuals would be instrumental in ensuring pre-emptive second and subsequent transplants for their patients. However, there is evidence that this is not the case and that more effort could be made to ensure a smooth transition to a pre-emptive second and subsequent transplant for those who are fit enough to receive one.

While there are good data on renal allograft failure rates in the U K through the adult and paediatric kidney specific report, published annually by NHS Blood and Transplant (NHSBT), there is a paucity of data on re-transplantation rates since they are not included in the UK Renal Registry (UKRR) annual report. The kidney specific report indicates that for adults in the UK, death censored renal allograft loss at 5 years is 13% for deceased donor renal transplant recipients (RTRs) and 7% for live donors, compared to 17 and 14% respectively in children [1]. In the USA, 13.1% of the adult waiting list was for re-transplantation in 2015, compared to 16.1% in 2005, compared to 16.9 and 24.6% respectively for the paediatric waiting list [2]. The actual re-transplantation rates performed were 13.2% (*n* = 2354) in adults and 8.5% in children (*n* = 186). The consideration for children and young people as they embark on their end-stage kidney disease journey is the risk-benefit profile of giving the best immunologically matched and good quality renal allografts as they may require multiple renal transplantation operations during their lifetime. This was emphasised by a Dutch study of 249 RTRs, all transplanted under the age of 15 years, which showed that after a mean follow-up of 25 years of follow-up, 36, 34, 17 and 5% had been transplanted two, three, four and five or more times, respectively [3]. Consequently, it is important to ensure that the pathway is as smooth as possible and to minimise the time spent on dialysis.

Adult RTRs with an estimated glomerular filtration rate (eGFR) less than 30 mls/min/1.73m^2^ contributed 13.3% of the UK transplant population in 2015 according to the 19th UKRR report [4]. This represents nearly 4500 patients with stages IV-T and V-T chronic kidney disease. Evidence from the UK Renal Registry demonstrates that the care of these patients is suboptimal. For example, across the UK, 16% of these patients have a haemoglobin less than 100 g/L, and less than a quarter achieved satisfactory blood pressure control (< 130/80 mmHg).

There was a high incidence of chronic kidney disease complications in our published cohort of 129 paediatric RTRs of whom 66% had stages III-T or IV-T chronic kidney disease [5]. There was evidence of treatment for hypertension in 53% (of whom 7% had uncontrolled hypertension), albuminuria in 60%, anaemia in 50%, acidosis in 39%, hyperparathyroidism in 20%, hypoalbuminaemia in 16%, hyperphosphataemia in 12% and hypocalcaemia in 3%. Interestingly, 30% of RTR were pre-emptively transplanted with their first transplant, and they had improved outcomes and reduced incidence of stage IV-T chronic kidney disease [6].

Data (extracted in October 2017 for 1007 adult RTRs) from our adult unit revealed 73 patients with an eGFR ≤ 20 mls/min/1.73m^2^. In this group, 19.2% had a bicarbonate under 20 mmol/L, 39.7% had a haemoglobin less than 100 g/l, 30.1% had a PTH greater than 30 pmol/l and only 30% (22/73) had been assessed for re-transplantation. Data from the USA described nearly two thirds of failing adult transplant patients commencing haemodialysis with intravenous catheters which is disappointing since they are nearly all under regular renal review [7]. These issues with patient care will be exacerbated by the continuing expansion of this population group which will make management increasingly difficult unless resources are allocated accordingly. As a result of the successful increase in deceased donor renal transplantation rates in the UK, the prevalent adult transplant population has increased from 19,074 in 2005 to 32,624 in 2015. To meet this challenge, some units have established transplant low clearance clinics to facilitate better management of this patient population. The recent UK Renal Registry report measured the evolution of renal function after transplantation with the median change in eGFR being − 0.56mls/min/1.73m^2^/year in adults. Interestingly, significantly faster rates of deterioration were described in RTRs who were younger, non-white, female or diabetic. These interesting findings warrant further investigation and may identify high risk sub groups. Renal transplant recipients whose transplant failed during the time period (*n* = 1306) had a median decline rate of − 6.3mls/min/1.73m^2^/year (IQR − 3.0 to − 12.1) which can be used to guide the process of working these patients up for further renal replacement therapy.

Outcomes after the failure of a transplant are generally poor. A meta-analysis of 40 studies in adults revealed that the mortality rate in the first 12 months following renal allograft failure was approximately 12% for the first 12 months dropping to around 6% for the next 3 years [8]. This is equivalent to the mortality rate a 70-year old first time starter on dialysis therapy in the UK. Even among the cohort who are fit enough relisted outcomes are inferior compared with transplant naïve patients, a recent study from Spain demonstrating a 50% increase in mortality [9]. A study derived from the Dialysis Outcomes and Practice Patterns Study (DOPPS) compared the outcomes in adult patients starting dialysis either for the first time (*n* = 2806) or after transplant failure (*n* = 1865) [10]. The patients with failing transplants had significantly higher mortality and hospitalisation rates, particularly related to infection. In addition, they had significantly lower quality of life scores and a significantly higher prevalence of depression. The psychological effects of renal allograft failure cannot be overstated, and detailed psychological studies have described the devastating effects of renal allograft failure, likening the process to a disenfranchised grief reaction (i.e. where the griever is not recognised as an individual who can and should grieve) [11]. Despite these observations, there is still data to suggest benefit of a second pre-emptive transplant with improved patient and graft outcomes [12, 13].

There is published guidance in the British Transplantation Society Guidelines for the Management of the Failing Kidney Transplant [14] and also the Renal Association Guidelines for the Planning, Initiating and Withdrawal of Renal Replacement Therapy [15]. These documents contain many sensible recommendations although it is conspicuous that the evidence levels are low. Implementation of these standards in the burgeoning renal transplant population is likely to present a considerable challenge. Realisation of guideline standards has unfortunately received far less attention than the diligent work that has gone into formulating them, and registry data reflects the fact that quite simple targets are frequently not achieved.

There is little evidence to guide the timing of dialysis initiation after renal transplantation. Seminal studies in patients with native renal failure have suggested that early initiation is not beneficial [16]. Limited data in RTR with failing grafts have revealed an association between higher eGFR at initiation and worse outcomes [17, 18]. It seems sensible that renal replacement therapy should be commenced for symptoms or fluid control.

Indications for graft nephrectomy are well established but it should be appreciated that there is a mortality rate of up to 1.5% at 30 days [19, 20]. There is evidence that graft nephrectomy is associated with a risk of sensitisation to both Human Leukocyte Antigens (HLA) classes I and II [21]. However, interpretation of this data is confounded by another of other factors:

- The indication for nephrectomy is often the result of a clinical syndrome caused by antibody-mediated rejection
- Peri-operative transfusion may occur
- Intracapsular nephrectomy leaves increased allogeneic material compared to extracapsular extraction
- Variation in immunosuppression withdrawal protocols around the time of nephrectomy
- Loss of the “sponge” effect as the graft no longer “mops up” antibodies

We have shown that 53% of paediatric RTR with renal allograft failure underwent transplant nephrectomy over a decade in our unit [22]. Grade II Banff rejection, an inflammatory response and early graft loss within the first year after renal transplantation are more likely to require transplant nephrectomy, which may be associated with higher circulating HLA antibody levels in paediatric RTR.

A randomised controlled trial of nephrectomy in asymptomatic patients is required to resolve this issue and to decide whether it is the nephrectomy per se, or the inflammatory milieu that leads to nephrectomy, which triggers sensitisation.

The correct strategy for weaning immunosuppression (IS) is not clear and there are conflicting priorities as shown in Table 1. Interpreting the outcome of IS withdrawal is complicated by concurrent nephrectomy which often occurs. There are no prospective randomised trials of IS withdrawal but several retrospective studies. Lachmann et al. examined the sensitisation of 54 adult patients who had failed renal transplants divided into three groups, Group 1 (*n* = 28, nephrectomy and IS withdrawal), group 2 (*n* = 14, nephrectomy only) and group 3 (*n* = 12, IS withdrawal only) [23]. In the first group, 75% (21 of 28) RTR had undergone nephrectomy in the first 6 months after transplantation, whereas groups 2 (median 1919 days post Tx) and 3 (median 3381 days post Tx) comprised a later vintage and presumably represented a different clinical phenotype. Their results suggested that nephrectomy was associated with a rise in donor specific anti-HLA class I antibodies, possibly due to the removal of adsorbing HLA class I antigens in the nephrectomy (“sponge” hypothesis). In contrast, the withdrawal of IS was associated with increasing titres of class II anti-HLA antibodies. Another important study from the Cambridge group demonstrated the association of increasing HLA mismatches with donor specific sensitisation, with each mismatch increasing the chance of developing donor specific antibodies by 41%, 1 year after relisting [24]. This finding underpins the importance of avoiding mismatches in any patient who is likely to require more than one transplant. They also observed that graft nephrectomy associated with an odds ratio of 3.42 for the development of DSAs. Interestingly, remaining on one or two (usually tacrolimus and MMF) immunosuppressive agents was associated with an odds ratio of 0.90 and 0.15 respectively for the development of subsequent DSAs. Taken together, these results suggest that if a second transplant is likely to occur within 12 months (e.g. live donor or high priority deceased donor renal transplant after early failure) then immunosuppressive therapy should be maintained. This is a complex clinical decision due to the infection risks on dialysis therapy and should be made according to individual circumstances by the transplant team in a timely fashion and documented before the initiation of renal replacement therapy.

**Table 1:** Advantages and disadvantages of continuing immunosuppression in renal transplant recipients with failing renal allografts

Advantages	Disadvantages
Less sensitisation	Malignancy risk
Less rejection	Infection risk
Maintain urine output	Cardiovascular risk
Avoid nephrectomy	Drug side effects

When patients are relisted for a second transplant, then they will often have a number of unacceptable antigens registered with NHS Blood and Transplant in the U K. These are based on the following categories:

- Antigens that correlate with anti-HLA antibodies detected in serum screening samples that are above a locally agreed threshold (mean fluorescence intensity – MFI)
- Antibodies that have previously been detected but are no longer present (historic antibodies)
- Antigens to be avoided since they are present in future potential live donors
- Antigens that were present on a previous graft that have never been detected in serum

Local practice varies, and it is essential to establish good communication with your local Histocompatibility and Immunogenetics (H&I) Laboratory in order to understand these processes. Most units in the UK are looking at less conservative strategies to increase the chances of transplantation in highly sensitised patients in the first two categories [25]. Further, scrutiny of the literature suggests that the last category may be unnecessarily conservative. It has been argued that any allogenic HLA antigen seen by the immune system represents “original sin”. Alternatively, it might be argued that if the relevant antibodies have never been detected then the recipient immune system is “regulated” not to respond. Away from the hypothetical arguments the experimental data does not support a strong effect. There have been many retrospective studies looking at repeat mismatches in second and subsequent transplants but a recent study merits discussion [26]. The authors looked at 13,789 adult recipients of second renal transplants carried out in the USA between 1995 and 2011, of which 3868 were had repeated HLA mismatches. Repeated mismatches of HLA class I antigens had no significant effect on any outcomes. Mismatches of HLA class II antigens had some mild effects particularly in those who had higher calculated reaction frequency values or who had undergone graft nephrectomy. This was the largest study of its kind and was carried out in the era of modern immunosuppression and H&I techniques. It is practically important since removing undetected class I mismatches and in some cases class II mismatches may considerably increase the chances of a patient receiving a second or subsequent transplant. More importantly, it emphasises the need of maintaining an ongoing dialogue with your H&I Laboratory.

Living donor transplantation has evolved considerably over the last decade with the establishment of the UK living donor sharing scheme and the burgeoning of both blood group incompatible and HLA-incompatible transplantation. This is important since some patients who are being evaluated for re-transplantation may well have been informed that potential donors were unsuitable at the previous time of consideration and it may worthwhile revisiting the subject of living donation. It is sensible to check blood group antibody titres (i.e. anti-A1 titres and anti-B titres in a blood group O recipient) since they can often be low. Our previous study suggested that approximately a third of children screened had titres of anti-A or anti-B antibodies that were 1 in 8 or lower [27]. This is a level which would facilitate relatively low risk ABO incompatible transplantation. If ABO incompatible deceased donor renal transplantation was available to children, then this could potentially increase the transplant activity by 2.2% and reduce the median waiting time by 21 days. Alternatively, pairs who were previously deemed incompatible may be considered for the living donor sharing scheme, and it is notable that this route now contributes more 10% of the overall live donor activity in the UK.

In summary, there is good evidence that the period around transplant failure is very traumatic for RTRs with a high incidence of physical and psychological illness. There is also a wide body of evidence to suggest that patient management is suboptimal at this time with relatively low achievement of simple targets laid out in guidelines. It is incumbent upon the transplant community to recognise and treat advanced CKD effectively in RTR and engage in shared decision making to choose options for further renal replacement therapy. This should include early consideration, education and preparation for a second renal transplant where appropriate.

## Self-assessment multiple-choice questions (answers are provided following the reference list)

Answer T (true) or F (false) for each:

1. According to United Kingdom Renal Registry data which of the following attributes is associated with more rapid progression in failing allografts?
  - Cause of native kidney disease
  - Diabetes mellitus
  - White race
  - Female sex
  - Young age
2. Regarding second and subsequent transplantation candidates
  - They are more likely to develop cancer
  - Should be weaned of immunosuppression
  - Comprise more than 10% of listed patients in the United States
  - Mortality rates are approximately 10% in the first year after graft failure
  - Comprise a higher proportion of the waiting list in adults than children in the United States
3. The period leading up to allograft loss is associated with which of the following?
  - Poor blood pressure control
  - Mineral bone disease
  - Higher prevalence of anaemia
  - Metabolic alkalosis
  - A high prevalence of pre-emptive vascular access placement
4. Which of the following factors are likely to increase sensitisation after failure of a renal transplant?
  - Episodes of late rejection
  - Nephrectomy
  - Blood loss and associated transfusion
  - Extra capsular technique of nephrectomy
  - Maintenance of dual immunosuppression
5. Which of the following is true related to transplant nephrectomy?
  - Up to 50% of children require graft nephrectomy
  - It is more likely after severe rejection
  - It is more likely after late, as opposed to early, renal allograft failure
  - Usually results in the removal of all allogeneic material
  - May result in new antibody detection due to loss of the “sponge” effect
